# Obstetric violence and associated factors among women who gave birth at public hospitals in Addis Ababa city administration, Ethiopia

**DOI:** 10.3389/fgwh.2024.1417676

**Published:** 2024-10-25

**Authors:** Getinet Tilahun Simeneh, Getaye Worku Tesema, Befikad Assefa Seifu, Nebiyou Tafesse, Abemelek Zegeye Hailemariam, Feruza Mehammed Suleyiman, Digafe Tsegaye Nigatu

**Affiliations:** ^1^ Health Service Quality Improvement Directorate, Gandhi Memorial Hospital, Addis Ababa, Ethiopia; ^2^ National Data Management & Analytic Center, Ethiopian Public Health Institute, Addis Ababa, Ethiopia; ^3^ Department of Nursing and Midwifery, St. Peter's Specialized Hospital, Addis Ababa, Ethiopia; ^4^ Department of Public Health, Menelik II Medical and Health Science College, Addis Ababa, Ethiopia; ^5^ Department of Public Health, Yekatit 12 Hospital Medical College, Addis Ababa, Ethiopia

**Keywords:** obstetric violence, women, labor and delivery, Ethiopia, respectful maternity care, childbirth

## Abstract

**Background:**

Obstetric violence during labor and delivery is one of the main reasons that women do not seek care from health caregivers in health facilities. Developing respectful maternity care services for women is the most important approach to ensure better newborn and maternal outcomes.

**Objective:**

This study aimed to assess the magnitude of obstetric violence and associated factors among women who gave birth at public hospitals in Addis Adaba city administration, Ethiopia.

**Methods:**

An institution-based cross-sectional study was carried out among 409 mothers who had given birth at two public hospitals (Gandhi Memorial Hospital and Abebech Gobena Mothers and Children's Health Hospital) in Addis Ababa, Ethiopia, from 1 to 30 May 2023. A systematic sampling method was applied and data were collected using a structured face-to-face interview questionnaire and entered into EpiData 3.1. The data were analyzed using Statistical Package for Social Science version 25. Bi-variable and multivariate analyses were performed. Statistical significance was declared at a *P*-value <0.05.

**Results:**

In total, 318 mothers [77.8% with a 95% CI (73.64–81.96)] had experienced obstetric violence in the study settings. Being more educated [Adjusted Odds Ratio (AOR) = 6.43; 95% CI 2.92–14.17], having ≥4 antenatal care contacts (AOR = 3.59; 95% CI 1.91–6.75), being multiparous (AOR = 2.65; 95% CI 1.32–5.32), induction of labor (AOR = 3.39; 95% CI 1.69–6.79), vaginal delivery (AOR = 0.25; 95% CI 0.11–0.62), and female birth attendants AOR = 2.42, 95% CI (1.31–4.47) were significantly associated with obstetric violence.

**Conclusion:**

More than three-fourths of the participants experienced obstetric violence. Thus, stakeholders need to develop interventions by taking all risk factors of obstetric violence into account.

## Introduction

Obstetric violence (OV) refers to a range of abusive or dehumanizing practices and behaviors that result in women’s loss of independence and the inability to make free decisions about their own bodies and sexual activities, and can occur during pregnancy, childbirth, or the postpartum period (1, 2). According to Brower and Hills, OV can be classified into seven forms: i.e., physical abuse, non-confidential care, non-dignified care, non-consented care, abandonment/neglected care, discrimination, and detention (3–5).

Globally, approximately 140 million births occur each year and those women have been advised to give birth in healthcare institutions to ensure access to skilled healthcare providers. However, accessing labor and delivery services in healthcare institutions may not be guaranteed due to various forms of OV including physical abuse, non-confidential care, non-dignified care, non-consented care, neglected care, discrimination, and detention in many facilities (3, 6–8).

Reducing maternal and child mortality is a worldwide concern, as stated in the Millennium Development Goals (MDGs) and the Sustainable Development Goals (SDGs), which aim to limit the maternal mortality rate to less than 70 per 100,000 live births and neonatal mortality to at least 12 per 1,000 live births (9). Maternal and neonatal mortalities are unacceptably high; 303,000 and 2.7 million mortalities occurred globally in 2015 respectively. Of these, almost all of the mortalities (99%) occurred in underdeveloped countries, including sub-Saharan Africa (SSA) (10).

OV is one of the silent contributing factors to both maternal and newborn morbidity and mortality, as many women experience mistreatment during labor and delivery in health institutions around the world. Furthermore, it is more prevalent in populations with limited resources, and it is also a factor in a woman’s choice of her next child’s birthplace (11).

According to the 2019 Ethiopia Mini Demographic and Health Survey (EMDHS), the prevalence of institutional delivery was low (48%) (12). Lack of respectful care, a fear of showing one’s body to health professionals, and the perceived cost of giving birth in a health facility were identified as factors contributing to low delivery rates in facilities (13).

When women experience OV from their healthcare givers during institutionalized childbirth, it violates the laboring women's right to respectful maternity care (RMC), putting their health, life, bodily integrity, and equality at risk (14).

The fear of these non-dignified and abusive treatments that women often experience during institutional-based childbirth is the main hindrance to utilizing skilled care rather than any other recognized barriers, especially in countries with high maternal mortality (15, 16).

In developing countries, including Ethiopia, the lack of RMC during labor and delivery continues to cause problems, as shown by the maternal morbidity and mortality rates (17, 18). The poor practice of RMC, i.e., obstetric violence during labor and delivery, leads to low healthcare facility-based births and results in slow progress in the attainment of an improved healthcare delivery system in the country (17, 19, 20).

In addition to these issues, there is limited research that assesses OV during institution-based childbirth in the study area.

Assessing the prevalence of these hidden and unacceptable practices during childbirth is a critical component for strengthening maternity services in Ethiopia and reducing maternal and newborn morbidity and mortality. Thus, this study aimed to assess the prevalence and associated factors of OV among women who gave birth at public hospitals in Addis Adaba city administration, Ethiopia.

## Methods

### Study design and period

The study employed an institutional-based cross-sectional design in two randomly selected public hospitals in Addis Ababa, i.e., Abebech Gobena Mothers and Children's Health Hospital (AGMCHH) (an affiliate of Yekatit 12 Hospital Medical College) and Gandhi Memorial Hospital (GMH) from 1 to 30 May 2023. Addis Adaba city administration has six public hospitals and each hospital has maternity services. Both the selected hospitals provide services such as family planning, antenatal care (ANC), labor and delivery, postnatal care, a maternal intensive care unit (MICU), comprehensive abortion care (CAC), cervical pre-cancer screening and treatment, infertility treatment, a fetal-maternal subspecialty clinic, immunization, a neonatal intensive care unit (NICU), a pharmacy, and a laboratory service. The hospitals are also staffed with intern doctors, midwives, resident doctors, obstetricians, and other healthcare workers and supporting staff.

### Source and study population

The source population was all the mothers who had given birth at Addis Adaba city administration public hospitals during the study period.

### Inclusion and exclusion criteria

Women who had given birth at GMH and AGMCHH and women who had completed their hospital stay just after a discharge decision had been made (i.e., to prevent the underreporting of OV during their hospital stay) during the study period were included in the study while women who had experienced a loss of consciousness or were unable to remember their labor and delivery process were excluded.

### Sample size determination

The minimum sample size required for this study was determined using both specific objectives and the largest sample size was taken to include all forms of OV. Thus, a single population proportion formula was used which considered the following assumptions: proportion of physical abuse (46.9%), non-consented care (63.6%), non-dignified care (55.3%), stigma and discrimination (9.3%), non-confidential care (32.3%), neglected care (12.7%), and no mothers who reported any form of detention in the healthcare facility. These figures were taken from a previous study conducted at the University of Gondar Comprehensive Specialized Hospital (17). The level of confidence was 95% and the margin of error was 5% (*d* = 0.05). The sample size for each form of OV was calculated as follows, and the largest number was used:

$$\mathrm{Sample}\;\mathrm{size}\;(n)=\frac{Z_{a/2}^{2}*P^{*}q}{d^{2}}$$

where *p* is the proportion of women who experienced obstetric violence during childbirth, *q* is the proportion of women who experienced no obstetric violence during childbirth, and *d* is the margin of error.

Thus, the final sample size was found to be 421 after allowing for a 10% non-response rate.

Sample size determination for the second objective, i.e., factors associated with OV, was calculated using OpenEpi by using some variables from previous studies (19, 21, 22) (Supplementary Table S1).

### Sampling technique and procedure

To include 421 women in this study, a proportional allocation method was used based on the number of women who gave birth at the two hospitals in the month preceding the data collection period. Thus, 208 and 213 women were enrolled from GMH and AGMCHH, respectively. A systematic random sampling technique was used for the enrollment of the 421 postpartum mothers who gave birth in the study area. Using the assumptions of *N* (the estimated deliveries in the two hospitals in a month) = 1,780 and *n* (the required minimum sample size including 10% non-response rate) = 421, we calculated the sampling fraction (*k*): where *k* = *N*/*n* ⇒ 1,780/421 ≈ 4. Thus, the study participants were selected using a sampling interval of 4.

### Dependent and independent variables

The outcome variable was obstetric violence and the explanatory variables included socio-demographic characteristics and provider-related, client-related, and obstetric factors.

### Operational definitions

Obstetric violence was defined as having occurred when the participants answered “yes” for at least one type or form of OV. Each form of OV has its own verification criteria, and a total of 25 verification criteria were used. Thus, the women who answered “yes” to at least one type/form/of OV were considered to have been victims of OV (3, 23) (Annex: verification criteria in the Supplementary Material).

### Data collection tools and procedures

Data were collected in face-to-face interviews using a structured questionnaire adapted from the WHO intrapartum care recommendations for a positive childbirth experience and other related literature (3, 23). OV was measured using seven performance standards or forms of violence including physical abuse, non-confidential care, non-dignified care, non-consented care, neglected care, discrimination, and detention using the verification criteria (a total of 25 criteria) developed by the Maternal and Child Health Integrated Program (MCHIP). The reliability of the tool was checked by computing Cronbach's alpha with a normal range value (0.8).

The questionnaire was first prepared in English, translated into the local language (Amharic), and then translated back into English by the principal investigator to check the consistency. Four midwives who provide free services outside the study facilities, along with two professional midwives, were recruited as data collectors and supervisors, respectively.

### Data quality assurance

A daylong training session was conducted for data collectors and supervisors (four data collectors and two supervisors) on the objectives and benefits of the study, individuals’ rights, informed consent, and data collecting techniques. Further pre-testing of the tool with 5% of the sample size, i.e., 21 mothers at Zewditu Memorial Hospital (one of the six hospitals in Addis Adaba city administration) which was not included in the study, was done. To prevent non-reporting or underreporting of OV during their hospital stay, postpartum mothers were interviewed just after they received their discharge decision.

### Data processing and analysis

Data was entered into EpiData version 3.1 and exported to the Statistical Package for Social Science (SPSS) version 25 software package for analysis. Before analysis, data processing (cleaning, counting, categorizing, and computing) was performed. Descriptive statistics were employed by computing summary statistics such as frequency, mean, percentages, and standard deviations. Assumptions of logistic regression were checked before analysis. The Hosmer–Lemeshow test was used to test the model's goodness of fit. Binary logistic regression was conducted to assess the crude relationship between the independent variables and the dependent variable. All variables with a *P*-value <0.25 were considered candidates for the multivariate logistic regression. Multivariate logistic regression was conducted to assess the independent effect of each variable on the outcome variable. The results were presented in the form of texts, tables, and/or figures. The degree of association between the variables was demonstrated by the odds ratio and significance level using a 95% confidence interval.

## Results

### Socio-demographic characteristics of the study population

A total of 421 postpartum women were invited, of which 409 were interviewed with a 97.1% response rate. The socio-demographic information provided by the respondents was not significantly different between the two hospitals. Of the respondents, 219 (53.5%) were aged between 25 and 34 years with a mean age of 27.58 years (±SD 4.85) and minimum and maximum ages of 19 and 40, respectively. Furthermore, 274 (67.0%) study participants were permanently living in urban areas and 371 (90.7%) of the mothers were married. Regarding the educational status of the participants, 218 (53.3%) had attended secondary school and above and 63 (15.4%) had no formal education. Finally, 192 (46.9%) participants were private employees whereas 23 (5.6%) mothers were merchants, students, or daily laborers by occupation (Table 1).

**Table 1:** Socio-demographic characteristics of the women who gave birth at public hospitals in Addis Ababa, Ethiopia, 2023 (*n* = 409).

Background characteristics	Category	Frequency (*n*)	Percentage
Age (years)	18–24	127	31.1
	25–34	219	53.5
	35–49	63	15.4
Residence	Urban	274	67.0
	Semi-urban	135	33.0
Marital status	Single	17	4.2
	Married	371	90.7
	Others^a^	21	5.1
Educational status	No formal education	63	15.4
	Primary education	128	31.3
	Secondary and above	218	53.3
Husband education status	Primary education	153	37.4
	Secondary and above	256	62.6
Occupation	Housewife	77	18.8
	Private employee	192	46.9
	Government employee	117	28.6
	Others^b^	23	5.6

^a^ Divorced and widowed.

^b^ Merchant, student, and daily laborer.

### Obstetric characteristics of mothers

Of the total number of respondents, 395 (96.6%) of mothers had ANC contact during their recent pregnancy, and among these, 280 (70.9%) had four or more ANC contacts and 218 (55.2%) had received it from midwives. Furthermore, 208 (50.9%) respondents were multiparous and 238 (58.2%) mothers gave birth through spontaneous vaginal delivery (SVD). For 142 (34.7%) of the respondents, labor was induced or augmented and 95% of the mothers (*n* = 218) had a previous history of institutional delivery (Table 2).

**Table 2:** Obstetric characteristics of the mothers who gave birth at public hospitals in Addis Ababa, Ethiopia, 2023 (*n* = 409).

Background characteristics	Category	Frequency (*n*)	Percentage
ANC contact	Yes	395	96.6
	No	14	3.4
ANC contact no. (*n* = 395)	<4	115	29.1
	≥4	280	70.9
ANC place (*n* = 395)	Health center	218	55.2
	Hospital	157	39.7
	Private health facility	20	5.1
ANC provider (*n* = 395)	GP and/or above	165	41.8
	Midwife	218	55.2
	Others^a^	12	3.0
Gravidity	Primigravida (G1)	167	40.8
	Multigravida (G2–4)	186	45.5
	Grand multigravida (≥5)	56	13.7
Parity	Primipara (*p*1)	191	46.7
	Multiparous (*p*2–4)	208	50.9
	Grand multiparous (*p* ≥ 5)	10	2.4
Previous institutional delivery (*n* = 218)	Yes	207	95.0
	No	11	5.0
Induction or augmentation of labor	Yes	142	34.7
	No	267	65.3
Mode or route of current delivery	Spontaneous vaginal delivery	238	58.2
	Assisted vaginal delivery	31	7.6
	Cesarean delivery	140	34.2
Time of delivery	Day	202	49.4
	Night	207	50.6
Blood transfusion	Yes	37	9.0
	No	372	91.0
Length of stay in hospitals (days)	<2	107	26.2
	≥2	302	73.8

ANC, antenatal care.

^a^ Nurse, health officer.

### Individual and provider-related characteristics

From the total respondents, almost three in five (249, 60.9%) of the mothers were not involved in decisions regarding their labor and delivery process, whereas half (50.4%) of the mothers normalized obstetric violence. More than half of the interviewed mothers, (215, 52.6%) were attended by female health professionals and three in seven of them (43.3%) were attended by midwives (Table 3).

**Table 3:** Individual and provider-related characteristics of mothers who gave birth at public hospitals in Addis Ababa, Ethiopia, 2023 (*n* = 409).

Background characteristics	Category	Frequency (*n*)	Percentage
Violence accepted	Yes	206	50.4
	No	203	49.6
Involved in decision	Yes	160	39.1
	No	249	60.9
Number of birth attendants	1–2	65	15.9
	3–4	148	36.2
	>4	196	47.9
Sex of the main birth attendant	Male	194	47.4
	Female	215	52.6
Profession of main birth attendant	Resident or obstetrician	167	40.8
	Midwife	177	43.3
	Intern doctor	65	15.9

### Prevalence and forms of obstetric violence

**Figure 1:**
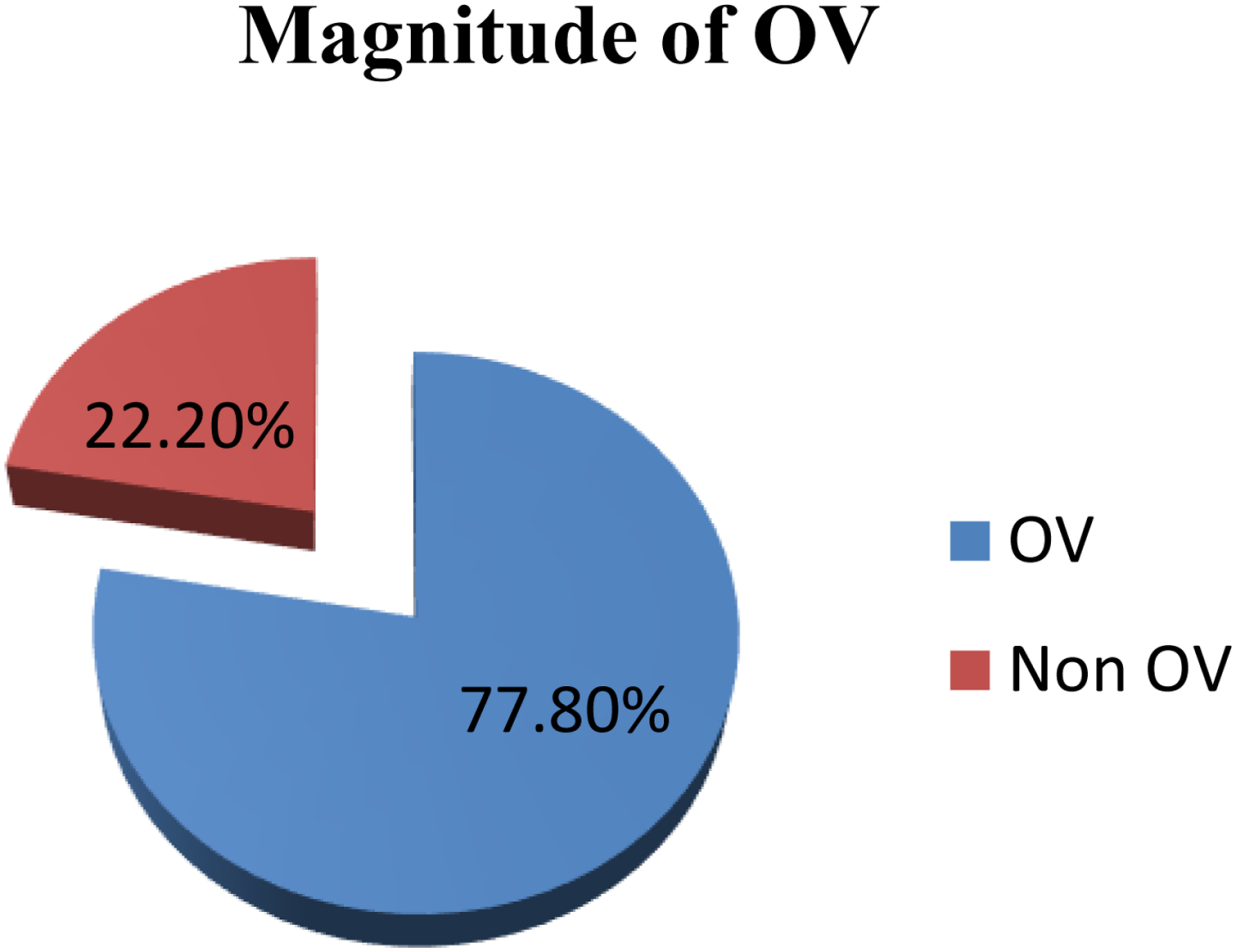
Prevalence of obstetric violence among mothers who gave birth at public hospitals in Addis Ababa, Ethiopia, 2023 (*n* = 409).

Among the 409 interviewed mothers, 318 [77.8% with a 95% CI (73.64–81.96)], reported having experienced at least one form of obstetric violence (see Figure 1).

**Figure 2:**
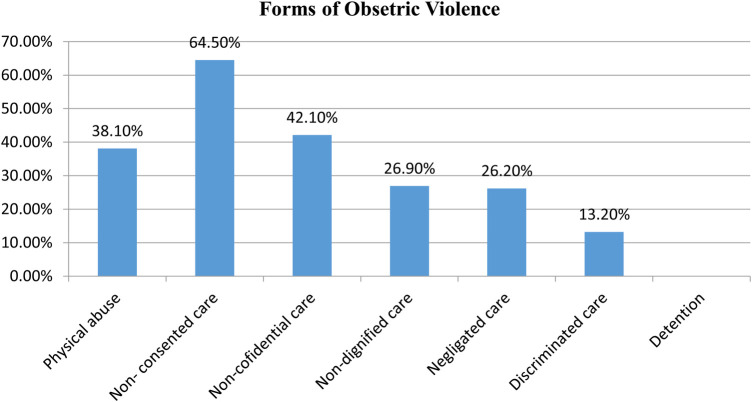
Percentage of each form of OV among mothers who gave birth at public hospitals in Addis Ababa, Ethiopia, 2023 (*n* = 409).

The most commonly experienced form of obstetric violence was non-consented care as 264 [64.5% with a 95% CI (59.7–69.3)] mothers reported having experienced it. This was followed by non-confidential care with 172 [42.1% with a 95% CI (37.3–46.9)] and physical abuse with 156 [38.1% with a 95% CI (33.4–42.9)] participants. Non-dignified care, neglected care, and discriminated care were reported by 110 (26.9%), 107 (26.2%), and 54 (13.2%) mothers, respectively, and no mother reported any form of detention in the healthcare facilities during childbirth (see Figure 2).

The most frequently reported criterion of OV was women not being allowed to give consent to healthcare providers before any procedure which was reported by 167 (40.8%) women. The healthcare provider not using curtains or other visual barriers to protect their privacy was reported by 148 (36.2%) mothers and the healthcare provider using fundal pressure was reported by 79 (19.3%). Furthermore, healthcare providers making negative comments toward them was reported by 54 (13.2%) participants, 87 (21.3%) reported healthcare providers ignoring them during labor while they were calling for help, and healthcare providers discriminating against them due to their socio-demographic status was reported by 47 (11.5%). These were reported in the domains of non-consented care, non-confidential care, physical abuse, non-dignified care, neglected care, and discriminated care (Supplementary Table S5).

### Factors associated with obstetric violence during institutional childbirth

In the bi-variable analysis, the age of the mother, educational status of the mother, number of ANC contacts of the mother, parity, induction or augmentation of labor, route of delivery, time of delivery, length of stay in the hospital, involvement in decisions, sex of main birth attendant, and profession of main birth attendant were associated with OV with *P*-values <0.25. Thereafter, in the multivariate logistic regression, a total of 11 explanatory variables with a *P*-value <0.25 in the bivariate logistic regression analysis were regressed against OV. As a result, in the multivariable logistic regression analysis, maternal educational status, ANC contact number, parity, induction or augmentation of labor, route of delivery, and sex of main birth attendant were significantly associated with OV at a *P*-value of <0.05.

Accordingly, mothers who had received a primary school education were two times more likely to report obstetric violence as compared to those who had no formal education [Adjusted Odds Ratio (AOR) = 2.42; 95% CI (1.10–5.35)]; while mothers who received secondary school education and above were six times more likely to report OV as compared to those who had no formal education [AOR = 6.43; 95% CI (2.92–14.17)].

Mothers who had four or more ANC contacts were 3.59 times more likely to report obstetric violence than their counterparts [AOR = 3.59, 95% CI (1.91–6.75)], whereas obstetric violence among multiparous mothers was 2.65 times higher than their counterparts [AOR = 2.65, 95% CI (1.32–5.32)].

The odds of experiencing obstetric violence among mothers whose labor had started with induction or augmentation were three times higher than those mothers who started labor spontaneously [AOR = 3.39, 95% CI (1.69–6.79)].

Mothers who delivered through cesarean delivery were 74.6% less likely to report obstetric violence as compared to women who gave birth through spontaneous vaginal delivery [AOR = 0.25, 95% CI (0.11–0.62)]. Finally, mothers who delivered with female birth attendants were two times more likely to experience OV as compared to mothers who delivered with male birth attendants [AOR = 2.42, 95% CI (1.31–4.47)] (Table 4).

**Table 4:** Factors associated with OV among mothers during institutional childbirth at public hospitals in Addis Ababa, Ethiopia, 2023 (*n* = 409).

Variables		Obstetric violence		COR with a 95% CI	AOR with a 95% CI
		Yes	No		
		*N* (%)	*N* (%)		
Age (years)	15–24	87 (21.3)	40 (9.8)	Ref.	Ref.
	25–34	177 (43.3)	42 (10.3)	1.94 (1.17–3.21)	1.02 (0.50–2.07)
	≥35	54 (13.2)	9 (2.2)	2.76 (1.24–6.13)	
Educational status	No formal education	29 (7.1)	34 (8.3)	Ref.	Ref.
	Primary education	96 (23.5)	32 (7.8)	3.52 (1.86–6.65)	2.42 (1.1–5.35)*
	Secondary and above	193 (47.2)	25 (6.1)	9.05 (4.74–17.29)	6.43 (2.92–14.17)***
ANC contact no.	<4	74 (18.7)	41 (10.4)	Ref.	Ref.
	≥4	233 (59.0)	47 (11.9)	2.75 (1.68–4.50)	3.59 (1.91–6.75)***
Parity	Primipara	138 (33.7)	53 (13.0)	Ref.	Ref.
	Multiparous	171 (41.8)	37 (9.0)	1.78 (1.10–2.86)	2.65 (1.32–5.32)**
	Grand multiparous	9 (2.2)	1 (.2)	3.46 (0.43–27.95)	5.32 (0.51–55.55)
Induction/augmentation/	Yes	127 (31.1)	15 (3.7)	3.37 (1.85–6.12)	3.39 (1.69–6.79)**
	No	191 (46.7)	76 (18.6)	Ref.	Ref.
Mode of delivery	SVD	193 (47.2)	45 (11.0)	Ref.	Ref.
	AVD	27 (6.6)	4 (1.0)	1.57 (0.52–4.72)	1.82 (0.48–6.81)
	C/S	98 (24.0)	42 (10.3)	0.54 (0.34–0.88)	0.25 (0.11–0.62)**
Time of delivery	Day	151 (36.9)	51 (12.5)	Ref.	Ref.
	Night	167 (40.8)	40 (9.8)	1.41 (0.88–2.25)	1.74 (0.96–3.15)
Length of stay in the hospital (days)	<2	78 (19.1)	29 (7.1)	Ref.	Ref.
	≥2	240 (58.7)	62 (15.2)	1.44 (0.87–2.40)	1.85 (0.89–3.83)
Involved in decision	Yes	113 (27.6)	47 (11.5)	Ref.	Ref.
	No	205 (50.1)	44 (10.8)	1.94 (1.21–3.10)	1.75 (0.90–3.39)
Sex of the main birth attendant	Male	139 (34.0)	55 (13.4)	Ref.	Ref.
	Female	179 (43.8)	36 (8.8)	1.97 (1.22–3.16)	2.42 (1.31–4.47)**
Profession of main birth attendant	Resident or obstetrician	128 (31.3)	39 (9.5)	Ref.	Ref.
	Midwife	134 (32.8)	43 (4.5)	0.95 (0.58–1.56)	0.68 (0.27–1.72)
	Intern	56 (13.7)	9 (2.2)	1.90 (0.86–4.18)	2.12 (0.67–6.75)

COR, crude odds ratio, Ref., reference; ANC, antenatal care; SVD, spontaneous vaginal delivery; AVD, assisted vaginal delivery; C/S, Cesarean delivery.

*P*-value: *<0.05; **<0.01; ***<0.001.

## Discussion

This study aimed to assess obstetric violence and its associated factors among women who gave birth in the selected health institutions. The study showed a high prevalence of OV as nearly four out of five [77.8% with a 95% CI (73.64–81.96)] of the women who participated reported having experienced at least one type of OV. OV was significantly associated with having received a secondary school and above education, ≥4 ANC contacts, being multiparous, labor started with induction, vaginal delivery, and being attended by a female birth attendant. The prevalence of OV in the current study is similar to other studies done in Ethiopia such as in South Wollo (79.4%) (3), Gondar University Comprehensive Specialized Hospital (75.1%) (17), and the Gedeo zone (79.7%) (19), as well as studies done in Mozambique (80%) (24), Italy (78.4%) (25), and Europe (76.3%) (26).

However, the prevalence of OV in this study is higher than a previous study conducted in Malawi (42.5%) (27) and a systematic review in SSA (44.09%) (28) as well as other previous studies in Spain (Part I, 38.3%) (29), Mexico (33.3%) (30), and Brazil (18.3%) (31). This finding is also higher than that reported in other studies conducted in Ethiopia such as in Tigray (22%) (32) and north Shewa (51.4%) (33), as well as the overall pooled prevalence of a systematic review and meta-analysis in Ethiopia (51.56%) (5). This difference might be due to variations in the types of data collection methods, study settings, and sample size (27, 28, 34). Unlike the current study settings (tertiary level), which had high case flows and many complicated referral situations, the majority of the aforementioned studies were conducted in low-level settings with limited client flow, which might mean healthcare providers are more likely to act abusively when there are too many clients and complicated problems. Other reasons might be due to differences in the study subjects, study period, and the timing of the interview after childbirth as in previous studies interviewees might have been affected by recall bias (29, 30, 34).

In contrast, the prevalence of OV in this study is lower than that reported in studies conducted in fourteen hospitals in Peru and Pakistan which showed a prevalence of 97.4% and 99.7%, respectively (35, 36). This difference might be due to the data in this study being collected through direct interviews with the mothers but in the previous studies, data was collected through direct observation. Hence, when data is collected through interviews, the prevalence of OV might be affected by the participants’ recall capacity and level of perception. The difference in prevalence could also be related to variations in the study settings and sample size. The current study's finding is also lower than that of studies done in Hawassa and in the North Showa zone of Ethiopia, both of which showed OV was experienced by all the study subjects (6, 37). This discrepancy might be attributed to the timing of the interview after childbirth and the verification criteria for the outcome variable that the researchers used. In this study, physical abuse was measured by six verification criteria while in the study done in North Showa, it was measured by 10 verification criteria. This study used as dependent variables the seven forms of OV women experience during facility-based childbirth but in the study done in Hawassa, Ethiopia, the domain of OV that was measured was different.

Regarding factors associated with OV during institutional delivery, mothers who had received education were more likely to report obstetric violence as compared to those who had no formal education (two times and six times more among mothers who had a primary school education and mothers who had a secondary school and above education, respectively). This finding is similar to studies conducted in Mexico and Nigeria, and other studies conducted in Ethiopia such as those in the Gedeo zone and at the University of Gondar Comprehensive Specialized Hospital (17, 19, 20, 30). This association could be due to the fact that educated women are more aware of their rights, are more perceptive, and thus more likely to report experiencing any sort of OV. It might be also as women become more educated, they become less accepting of any form of mistreatment during labor and delivery as a normal procedure and thus they become confident enough to report it (30, 38).

This study showed that as the number of ANC contacts increased, mothers were more likely to report OV. Accordingly, women who had four or more ANC contacts were 3.6 times more likely to report OV than their counterparts. However, this finding is opposed to studies conducted in Addis Ababa and Assosa, Ethiopia (22, 39), and the variation might be related to the data collection methods and study subjects’ differences. The association in this study could be related to the fact that women with a higher number of ANC contacts can easily ascertain the standard of birth care, are informed of their rights, and have an improved level of awareness of how to report the occurrence of any form of OV. However, even if more ANC contacts are typically encouraged and are linked to better health outcomes for both women and their babies, this may not be guaranteed due to systemic and contextual problems, such as frequent rushed interactions between healthcare providers, which can exacerbate power imbalances and cause them to feel abused (7).

The study has also found that multiparous mothers were at 2.65 times higher risk of experiencing OV than their counterparts. This is similar to studies conducted in Kenya and four regions of Ethiopia (16, 21) and in contrast with the study done in North Showa, Ethiopia (33). This association might be due to the fact that multiparous women have already given birth and are thus more aware of their rights and the standards of obstetric care and are therefore more likely to report OV. Another finding in this study was that OV among mothers whose labor had been started with induction or augmentation was more than three times higher than among those who started labor spontaneously. This might be because inducing labor is related to the risk of failure of induction, the requirement of more medical intervention compared to spontaneous vaginal delivery such as instrumental delivery, the need for cesarean delivery, and the fear of additional obstetric complications such as uterine rupture, postpartum hemorrhage, infection, and neonatal loss (40–42).

Moreover, mothers who delivered through cesarean section were 74.6% less likely to experience obstetric violence as compared to those women who gave birth through spontaneous vaginal delivery. Studies in Peru and the Hadiya zone of Ethiopia shared similar results (10, 35). Even though indications for cesarean section were not included as part of the data in this study, the reason might be that mothers who deliver through a cesarean delivery have fewer procedures than those who give birth vaginally, as the latter may experience repeated vaginal examination, prolonged labor, other birth-related complications, and an episiotomy without anesthesia.

In contrast, other studies showed that mothers who gave birth through SVD were less likely to experience OV compared to those women who gave birth through cesarean delivery (18, 43). This might be due to the fact that mothers who had a cesarean birth are more likely to have postpartum pain and a more difficult and longer recovery.

Finally, the results of this study also revealed that mothers who delivered with female birth attendants were also two times more likely to face OV as compared to mothers who delivered with male birth attendants. This finding is in line with the studies conducted in Hawassa and Addis Ababa (22, 37). The finding may be because male providers were more commonly considered to participate in RMC activities than female providers (44).

### Limitations of the study

Since it was a cross-sectional study design, it is difficult to establish temporal relationships between the explanatory variables and the outcome variable.

## Conclusion

The prevalence of obstetric violence was high in the study setting and was significantly associated with having a secondary school and above education, having four and more ANC contacts, being multiparous, induction or augmentation of labor, vaginal delivery, and a delivery attended with female birth attendants.

The Ministry of Health needs to strengthen initiatives to reduce obstetric violence by incorporating healthcare providers’ attitudes toward respectful maternity care and considering all risk factors. Healthcare providers should handle all mothers with compassion and a respectful manner, taking into account that mothers are different regardless of their mode of delivery and parity. The continuous provision of education and counseling throughout their ANC follow-up should be strengthened and awareness raised to increase the number of ANC contacts. Furthermore, maternity care providers should prioritize reducing OV among mothers whose labor had been started with induction or augmentation by providing proper information about the indication of inducing labor, its risks, and its complications. Female birth attendants have to meet respectful maternity care standards. We recommend additional research using a mixed approach in different study settings.

## Data Availability

The raw data supporting the conclusions of this article will be made available by the authors, without undue reservation.
