# Supplementary material for: Obstetric violence and associated factors among women who gave birth at public hospitals in Addis Ababa city administration, Ethiopia
Source: Front Glob Womens Health. 2024 Oct 25;5:1417676. doi: 10.3389/fgwh.2024.1417676 (PMC11543569; doi:10.3389/fgwh.2024.1417676)
Supplement: Supplementary file 1 [file Table1.docx]

# Supplementary information

*Table 1:- Sample size calculation for association factors of OV among women who gave birth at public hospitals of Addis Ababa city administration, Addis Ababa, Ethiopia, 2023:*

| S/n | Factors | Assumptions | | | | | | |
| --- | --- | --- | --- | --- | --- | --- | --- | --- |
|  |  | Ratio | Power | CI | Magnitude OV among exposed | Magnitude of OV among non-exposed | AOR | Total sample size |
| 1 | ANC follow up | 1:1 | 80% | 95% | 200 | 200 | 2.994 | 400 |
| 2 | Facing complications | 1:1 | 80% | 95% | 182 | 182 | 3.1382 | 364 |
| 3 | Parity | 1:1 | 80% | 95% | 159 | 159 | 3.36 | 318 |

*Table 5:- Types of obstetric violence by its verification criteria among mothers who gave birth at public hospitals of Addis Ababa city administration, Addis Ababa, Ethiopia, May 2023: (n=409).*

| Types of obstetric violence | | Experience/ frequency/ of obstetric violence | |
| --- | --- | --- | --- |
|  |  | Yes % | No % |
| **Physical abuse** | Physically hit or slapped | 18 (4.4) | 391 (95.6) |
|  | Separate the mother from her baby without medical indication | 70 (17.1) | 339 (82.9) |
|  | Restricted the mother from food or fluid without medical indication | 31 (7.6) | 378 (92.4) |
|  | The birth attendant used fundal pressure. | 79 (19.3) | 330 (80.7) |
|  | The mother receives unnecessary painful treatment/medication/ | 48 (11.7) | 361 (88.3) |
|  | The care provider touched the mother while she has been refusing to touch. | 78 (19.1) | 331 (80.9) |
| **Non-consented care** | Care providers did not introduce themselves and didn’t greet woman | 153 (37.4) | 156 (62.6) |
|  | Care givers did not encourage the woman and her companion to ask | 107 (26.2) | 302 (73.8) |
|  | The providers didn’t respond politely while a woman asks them | 84 (20.5) | 325 (79.5) |
|  | The providers did not explain what was being done during labor process. | 159 (38.9) | 250 (61.1) |
|  | Care providers did not obtain consent or permission before any procedure. | 167 (40.8) | 242 (59.2) |
| **Non-confidential care** | Did not use drapes or covering to protect the mother’s privacy | 148 (36.2) | 261 (63.8) |
|  | Disclose a mother’s medical information in a condition that others hear. | 71 (17.4) | 338 (82.6) |
| **Non-dignified care** | Shouted at or scolded the mother during labor. | 38 (9.3) | 371 (90.7) |
|  | Made negative comments to the mother during labor | 54 (13.2) | 355 (86.8) |
|  | Insult the mother and her supporters | 44 (10.8) | 365 (89.2) |
|  | Providers demonstrating the culturally inappropriate way | 23 (5.6) | 386 (94.4) |
| **Neglected/ Abandonment of care/** | A care provider ignored a mother when she called them to help | 67 (16.4) | 342 (83.6) |
|  | A woman was alone while she was in labor at a health institution | 87 (21.3) | 322 (78.7) |
|  | A mother experienced a life-threatening condition because no one helped her | 46 (11.2) | 363 (88.8) |
| **Discriminated care** | A care provider discriminated against mothers either by ethnicity, religion, or others | 47 (11.5) | 362 (88.5) |
|  | A care provider discriminated against mothers by medical status | 5 (1.2) | 404 (98.8) |
|  | Discriminated because of being a teenager. | 0 (0) | 409 (100) |

**Annex: Verification criteria**

The following lists are the seven forms of obstetric violence with their respective verification criteria and a woman who answers yes to at least one criterion then she was considered as being violated at the time of childbirth.

**Physical abuse**: Women who faced physical harm or ill-treatment. Measured by 6 verification criteria including 1. A health care provider physically hit or slapped. 2. A healthcare provider separates the mother from her baby without medical indication. 3. A healthcare provider restricted the mother from food or fluid throughout her labor without medical indication. 4. The birth attendant used fundal pressure. 5. The mother receives unnecessary painful treatment. 6. The care provider touched the mother while she refused to touch her.

**Stigma and discriminated care**: If a woman received no equitable care; measured by 3 criteria. 1. A care provider discriminates against them either by ethnicity, religion, or others. 2. A care provider discriminated by medical status. 3. A Healthcare provider discriminated against because of being a teenager.

**Abandonment of care:** A woman did not get care timely; measured by 3 criteria. 1. A care provider ignores a mother when she calls them to help. 2. A woman was alone while giving birth in a health institution. 3. A mother experienced a life-threatening condition because no one helped her.

**Non-dignified care**: A woman who is not treated with dignity and respect; measured by 4 criteria; 1. Providers shouted at or scolded the mother during labor. 2. Providers made negative comments to the mother during labor. 3. Staff insult the mother and her supporters. 4. Care providers demonstrating culturally inappropriate ways.

**Non-consented care**: women’s right to information is not protected; Measured by 5 verification criteria including 1. The care providers did not introduce themselves and didn’t greet the woman and her support. 2. The caregivers did not encourage the woman and her companion to ask them. 3. The providers didn’t respond politely when a woman asked them. 4. The providers did not explain what was being done during the labor process. 5. Care providers did not obtain consent or permission before any procedure.

**Non-confidential care**: A woman’s confidentiality and privacy were not protected, measured by 2 criteria 1. The provider did not use drapes or other coverings to protect the woman’s privacy during the procedure. 2. Healthcare providers disclose a mother’s medical information in a condition that others hear.

**Detention in facilities**: A woman was confined against her will; measured by 2 criteria 1. Discharge was postponed until hospital bills were paid. 2. A woman was detained in a health facility against her will.

***Note*: -** If a woman answers ‘yes’ to at least one criterion of the above verifications will considered to have OV at the time of childbirth.
